# Cost-effectiveness of breast, lung, colon, prostate and cervical cancer outcomes in Brazil: a worldwide comparison

**DOI:** 10.3332/ecancer.2021.1243

**Published:** 2021-06-07

**Authors:** Rodrigo Pellegrini, Tomás Reinert, Carlos Henrique Barrios

**Affiliations:** 1 Pontifícia Universidade Católica do Rio Grande do Sul (PUCRS), School of Medicine, Av Ipiranga, 6681, Partenon, Porto Alegre, RS, 90619-900, Brazil; 2 Latin American Cooperative Oncology Group (LACOG), Av Ipiranga, nº 6681, Prédio 99ª, Sala 806, CEP 90619-900, Porto Alegre, RS, Brazil; 3 Oncoclínicas, Av Praia de Belas, 1212, Sala 1606, Menino Deus, Porto Alegre, RS, 90110-000, Brazil

**Keywords:** cancer, efficiency, delivery of health care, incidence, mortality

## Abstract

**Introduction:**

Cancer is the second leading cause of death in the world, and it is expected to be the main cause by the year 2030. Current trends of higher incidence and the introduction of new treatments lead to the challenge of treating more people with increasing costs per capita. In Brazil, current and future challenges are even more significant because of the limited resources destined for healthcare.

**Methods:**

We propose a methodology to compare cost-effectiveness performance with a regression of cancer lethality against the resources available for different nations, using the gross domestic product and the mortality-to-incidence ratio. Our objective is to evaluate and compare outcomes observed in Brazil.

**Results:**

According to our methodology, Brazil is performing well in breast and prostate cancer (observed lethality 9% and 15% lower than expected, respectively). It performs close to expected in colon (0.8% higher) and cervix (2% higher). However, lung cancer had a higher lethality than expected (6.5% higher). We also found that breast, prostate and cervical cancers are the primary sites more related to income. Lung cancer had the weakest relationship with resources.

**Conclusion:**

Brazil has different cost-effectiveness results in the management of cancer depending on the primary site. Also, national income has a significant and heterogeneous effect on the lethality of different tumour types. This economic analysis is important for low- to middle-income countries seeking to evaluate cancer outcomes in limited-resource settings.

## Introduction

Cancer is one of the leading causes of death both for high-income countries (HIC) and low- to middle-income countries (LMIC). The estimated incidence for 2018 was 18 million cases with an associated mortality of 9.5 million deaths [1]. With the progressive growth and ageing of the population in association with an increasing prevalence of the main risk factors for cancer, this trend is expected to continue.

It should be recognised that there are significant disparities across different countries. In the USA, the death rate for cancer was 215 per 100,000 in 1991, falling significantly to 159 per 100,000 in 2015. The American Cancer Society credits these impressive results to the reduction of smoking, the implementation of screening and early diagnosis programmes and the development of newer and better treatment modalities [2, 3]. Although having a lower incidence of cancer, Latin America has a more significant cancer mortality burden than the USA or main European countries. All cancer mortality-to-incidence ratio (MIR) is 0.59 in Latin America, while it is much lower in the USA (0.35) and the European Union (0.43) [3]. Multiple factors have been proposed to explain these worse outcomes. Among them, poorly organised and fragmented health care systems and limited resources destined for cancer treatment are commonly cited [3].

At the same time, there are evident differences related to access to new treatments. The high cost of new drugs represents a significant barrier compromising availability in resource-limited scenarios. In 2014, the median cost of any new oral anticancer agent ($135,000 a year) was six times greater than in the early 2000s [4]. While access is a universal problem, in Brazil, an upper-middle-income country, but with an income per capita close to one-sixth the one in the USA and one-fourth of the one in the EU, this problem clearly generates much more concern (see the Appendix for LMIC and HIC current definition) [5, 6].

In a setting of scarce resources, LMIC need to develop public health policies with established cost-effectiveness. To achieve that, it is also important to measure each country’s performance in the management of cancer patients considering its available resources.

## Methods

Our objective with this ecologic study is to measure the outcomes of cancer management in Brazil, given its level of income, and compare it with other nation’s outcomes.

### Variables

As a measure of outcome, the MIR is a well-known proxy for the lethality in cancer patients [3, 7–9]. It is calculated by dividing the number of deaths by the incidence of a specific cancer type during a certain period. This ratio gives us the percentage of patients who will die from cancer (lethality), while the remaining share represents all the patients cured with treatment (survival ratio = 1 − MIR). It must be noted that both early diagnosis and better treatment have an impact on this ratio, reducing the number of deaths in relation to the total number of cases.

To estimate each country’s income restriction, we used the gross domestic product per capita (GDPpc). In economics, the GDPpc represents the whole production of a country divided by the number of citizens. Therefore, it has the same value as the median income per person (with taxes). Important to note in our study, because of the different characteristics of health care systems, the government expenditure per person is also considered in this calculation, resulting in the total amount of private and public expenditures. Therefore, with a regression of the MIR (as outcome) by the GDPpc (resources restraint), we can simulate a regression of cancer lethality by resource availability, estimating each country’s cost-effectiveness in dealing with cancer [10].

### Data

In order to create the regression, we used data from two different sources. The cancer incidence and mortality data for 2018 for each country were acquired from the International Agency for Research on Cancer, an institution of the World Health Organization (WHO) [11]. We used the age-standardised incidence rate and age-standardised mortality rate rather than the crude rates to diminish the impact of the populations’ heterogeneity. We selected data with an estimated error limited to 15% (already calculated by the WHO) to give more credibility to our analysis.

The GDPpc was acquired from the World Bank database, also for the year of 2018. We had two possible alternatives for GDP selection. GDPpc with current dollars or GDPpc with purchasing power parity. As different countries in the world have different inflation and exchange rates, the first option seemed more reliable to denote their citizens’ wealth.

The selected cancer sites are those responsible for most cancer deaths in women (breast, lung, colon and cervix) and men (lung, prostate and colon) in Brazil [12].

### Model

To address the comparison among different countries, we propose a regression of the MIR (as a measure of performance) of different countries against their GDPpc (a measure of resource restraint).

The regression was performed with an ordinary least squares (OLS) methodology, resulting in an equation reflecting the impact of GDPpc in the MIR for each primary site. Because of the exponential distribution of GDPpc across the world, we transformed the data to the logarithmic form.

This model generates three variables of interest: (i) Residues; (ii) Elasticities and (iii) R-squared (see Table 1 for description).

### Primary endpoint

The residues for Brazil are the primary endpoint of this article. They represent, for each primary tumour, how each country is performing in the management of that cancer. If the residue is positive, it means the lethality is higher than what the regression indicates it should be, given its GDPpc (expected value).

### Secondary endpoints

The elasticities and R-squared (R^2^) values will also be analysed. Each primary site generated one elasticity and one R^2^. The elasticity represents how much the lethality of a tumour decreases when income doubles. The R^2^ value represents how much of the variation of a tumour’s lethality between countries can be explained by their income variation.

## Results

### Data

Cancer incidence and mortality data from GLOBOCAN have 186 measurements. However, for each cancer site, we excluded countries with an estimated error of 15% or more. In general, most of the excluded data came from low-income countries. After this first screening, we matched the MIR (dividing each country’s cancer mortality rate by its incidence) with the GDPpc from each country (some of them were not available for the year 2017, e.g. Ven ezuela). The Brazilian data had estimated errors lower than 15% for all cancer sites. Data were available from 76 countries for breast cancer, 82 for lung, 68 for colon, 68 for prostate and 56 for cervix.

### Regressions

**Figure 1.**
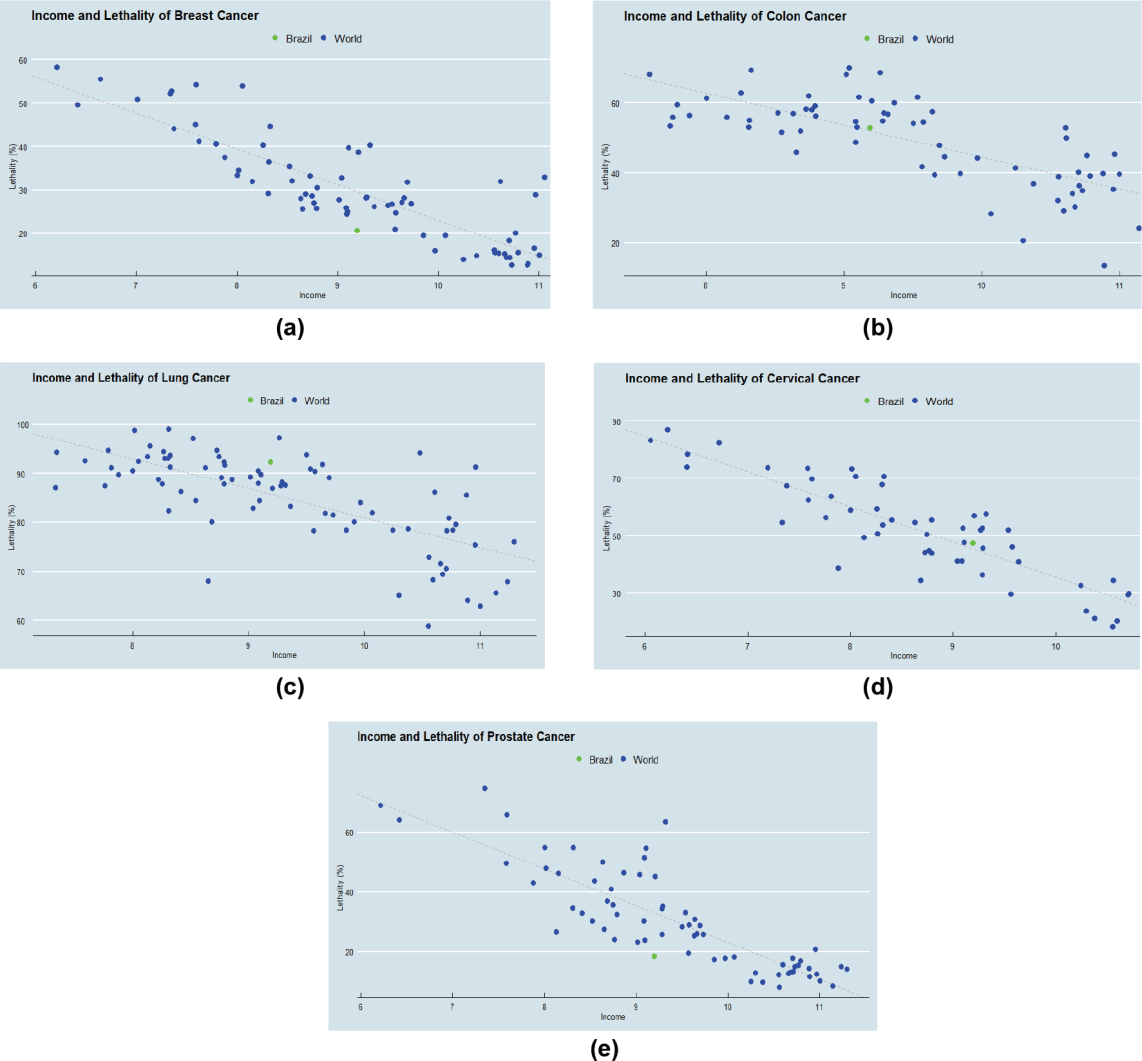
(a–e): Graphics for each cancer primary sites with data on the lethality (MIR) and income (GDPpc). Each point indicates one single country, and Brazil is indicated in green. When the green point (Brazil) is below the regression line in the graphic (such as for breast and prostate cancer), it indicates that the lethality of this primary site in Brazil is lower than it is in countries with similar income per capita. However, when the green point is above the regression line in the graphic (such as for lung cancer), the Brazilian lethality is higher than for countries with similar income per capita. Finally, when the green point is on the regression line (such as for colon and cervical cancer), results for Brazil are equal to the mean of similar income countries.

The regressions resulted in the graphics shown in Figure 1a–e (their equations are presented in the Appendix).

### Brazil’s outcomes

The real lethality and the predicted lethality for each primary cancer site for Brazil are represented in Table 2, as well as their difference. The lethality of breast and prostate cancer in Brazil is lower than expected (by an absolute of 9% and 15%, respectively). On the other hand, the lethality of lung cancer is higher than expected (6.5%). Colon (0.8%) and cervical cancer (1.9%) had results close to the expected (Table 2).

### Elasticities

The regressions’ elasticities (the proportion of decrease in MIR with the increase of GDPpc) indicate that income has more impact on the lethality of prostate and cervical cancer. An increase of 100% of a country’s income is expected to decrease the lethality of these primary sites by 8.5%. For lung cancer, the impact is the lowest (4.1%). Breast and colon cancer had intermediate values (5.7% and 6.3%, respectively).

### R-squared

The R^2^ values (the percentage of the difference in MIR across countries that their difference in GDPpc can explain) indicate that breast, lung and cervical cancer are the tumour sites with the lethality variation best explained by the variation on available resources (75%, 71% and 78%) (Table 3). On the other hand, the variation of lung cancer lethality is explained only 47% by the variation in income across the countries. Colon cancer had an intermediate result (57%).

## Discussion

We achieved our objective of evaluating the outcomes of common cancer subtypes in Brazil in relation to its available resources, and we observed that breast and prostate cancer results indicate a good cost-effectiveness performance. Conversely, lung cancer had a higher-than-expected lethality given the Brazilian GDPpc. Also, we obtained elasticities and R-squared results for each cancer site, representing a measure of the worldwide impact of income, suggesting that breast, prostate and cervical cancer lethality were all well explained by the model (as indicated by their high R-squared) and are strongly impacted by the increase in the availability of resources (as indicated by their high elasticities). However, lung cancer had both the lowest elasticity and R-squared [13, 14].

This analysis is important in the current context of increasing cancer incidence as well as cancer treatment costs. This scrutiny has a different impact across nations, mainly due to the worldwide variation on wealth, which is an already known cause for the difference in outcomes between HICs and LMICs [15–17].

In this ecologic study, we used available data from the World Bank and the WHO to evaluate the relation of GDPpc (as a measure of available resources) and the MIR (as a measure of cancer outcomes) with a particular focus on the results for Brazil. With this regression, we were able to analyse if the lethality of breast, lung, colon, prostate and cervix cancer in Brazil was compatible with its level of income. Also, the two other results generated by the model (elasticities and R^2^) are measures of the relationship between GDPpc and MIR data for each primary site.

Our analysis showed that the lethality of breast cancer in Brazil is 20%, while the country’s GDPpc would suggest a lethality of 29%. Its R^2^ demonstrates that income is a good predictor for breast cancer lethality, with 75% of MIR variation being explained by GDPpc. This data is compatible with the results of the literature on this subject [16]. We list two possible factors that could explain the better results of breast cancer patients in Brazil: the national screening programme and the adoption of high-quality treatments by the public health care system. Regarding screening, the Brazilian Ministry of Health indicates in its national guideline that all women 50–69 years should have periodic mammography with an interval of 2 years [18]. When we look at the staging at diagnosis data, the AMAZONA project indicates that 68% of breast cancer cases present in stages I and II in Brazil [19]. Even though these numbers could be improved and are lower than those observed in the USA, when we compare them to the data of a Lancet meta-analysis for African regions (West Africa = 23.35%; East Africa = 22.69%; Southern Africa (Black) = 22.32%; Southern Africa (White) = 47.7%), we consider them a reasonable outcome [20]. As for the quality of the Brazilian health care system’s treatment of breast cancer, since 2012, trastuzumab is offered at no cost to patients in the adjuvant setting. In metastatic disease, Trastuzumab approval for Sistema Único de Saúde (SUS, the Brazilian public health care system) patients came only in 2017 [21]. The system is organised with central purchases: the government makes big purchases each year (given the new-cases estimate), and then distributes the drug according to demand. This well-organised system may be considered an accomplishment, considering that it is a challenge to offer high-priced treatments even in nations with higher income per capita [22].

The observed lethality of lung cancer was higher than expected considering the national income in Brazil by 6.5%. However, data suggest that the relationship between lung cancer outcomes and wealth is weaker than for other primary sites. GDPpc can explain only 47% of the MIR variation, and a decrease of 4% in MIR is expected with a one-fold increase in GDPpc. This conclusion is in accordance with the results found in a meta-analysis published by Finke *et al* [14] on the relationship between lung cancer survival and socioeconomic differences. This higher lethality of lung cancer in Brazil requires careful analysis. Published data shows that lung cancer has a weaker association between income and survival. Despite good treatment options for some patients, survival is still relatively low. In virtue of the aggressiveness of this disease, it seems that currently available treatments do not have a significant impact on survival ratios (the 5-years survival rate in the USA is no more than 20%). Additionally, even in HIC, the disease is usually diagnosed in the advanced stage setting. SEER data shows that almost 80% of lung cancers are diagnosed already with locoregional or distant disease. The recent availability of immunotherapies that have revolutionised the management of advanced lung cancer and that may significantly extend the survival of some patients may play an important role in future analyses as the associated high costs will compromise access in LMIC.

In prostate cancer, it is well documented that incidence is higher and mortality is lower in HICs in comparison with LMICs [15]. Our findings are in accordance with this statement, as 71% of the variation on MIR could be explained by GDPpc, and a decrease of more than 8% is expected when GDPpc doubles. In Brazil, the observed lethality was found to be 15% lower than the predicted model (33% versus 18%). In prostate cancer, overdiagnosis and overtreatment are factors to consider in view of the indolent nature of the disease. The good performance in our model may be explained by confounding factors such as higher rates of incidence. Of note, the Brazilian incidence of prostate cancer (82 per 100,000) is higher than in Argentina (53), Chile (72), Colombia (52) and Peru (46). Furthermore, 70% of prostate cancers in Brazil are diagnosed as localised disease [11].

For cervical cancer, both incidence and mortality are lower in HICs than in LMICs. Considering that defined prevention strategies are widely available for this cancer, we should note there are significant differences in implementation between countries [17]. Our results found that GDPpc explains 78% of MIR data (the highest of all primary sites) with an elasticity of 8.4%. In our analysis, the observed lethality of cervical cancer in Brazil is close to the result predicted by the model (45% versus 47%).

The epidemiology of colon cancer is facing a transition. Developed countries are seeing a reduction in both incidence and mortality rates, while developing countries deal with an increase in both incidence and mortality rates [23]. Our results demonstrate intermediate values of elasticity (6.3%) and R^2^ (57%) – not as high as for breast, prostate and cervix, and not as low as lung cancer. The Brazilian result indicates that the observed lethality is in accordance with the value predicted by the model (52% and 51%).

This study can be a helpful tool for policymakers to decide how to best allocate resources in a scenario of limited availability. Results of elasticity and R-squared indicate that some tumours (with higher values) may be more sensible and impacted by the increase in resource application. We can conclude that investing in early diagnosis and the treatment of such primary sites may be more cost-effective than in others. For LMIC, this analysis is crucial given the current situation of increasing cancer incidence and costs. In our analysis, as previously demonstrated, breast, prostate and cervical cancer have stronger relationships with income than lung cancer [15–17].

Our analysis has limitations. There is no absolute indicator for the management of cancer. We used the MIR as the outcome proxy because it represents the lethality of cancer. It must be noted that not only the treatment efficacy impacts this indicator but also early diagnosis since more advanced cancer has a worse prognosis. This is the reason why we used the term outcomes of the management of cancer and not treatment. In addition, if we used the mortality rate isolated as our indicator, we would not be able to compare countries with different incidence rates. With MIR, we have not this problem.

We conducted this study using only income per capita as our main regressor and chose not to use factors such as the Human Development Index (HDI), the Gini index, the education level, the expenditure on health and the number of doctors. The reason was to focus on a simpler model with the most reliable data since the GDP is a better indicator with a more uniform calculation method than expenditure on health, mainly due to the different types of health care systems across the world. Since we used the GDP as the regressor, both public and private wealth are represented as resources available. In accordance with previous studies, we believe that GDP can be considered as one of the main factors to explain differences between countries’ cancer outcomes [16].

Equally important, the quality of the basic data needs careful consideration. To address this factor, we used published data from the WHO and the World Bank.

## Conclusion

Careful analysis of real-world data allows for better assessment of some observed disparities in cancer outcomes between HIC and LMIC. Strategic planning and resource allocation, particularly in limited resource scenarios, could benefit from this exercise. This analysis should be considered dynamic and subject to change as new therapeutic modalities become available, changing treatment outcomes for given tumour types. According to our results, while breast and prostate cancers have a good cost-effective performance in Brazil, in contrast, lung cancer has a worse than expected outcome, although it had the weaker relation of income and lethality of all tumours. In addition, investing in the diagnosis and treatment of certain specific tumours generates a higher impact on outcomes.

## Funding

No funding was received.

## Conflicts of interest

Rodrigo Pellegrini:

- None

Tomás Reinert:

- Speaker honoraria: Novartis, AstraZeneca, Pfizer, Libbs, Lilly, Pierre Fabre
- Consulting or advisory role: AstraZeneca, Lilly, Novartis
- Research funding: AstraZeneca

Carlos Henrique Barrios:

- Stock and other ownership interests: Biomarker, MedSIR, Tummi
- Speaker honoraria: Novartis, Roche/Genentech, Pfizer, GlaxoSmithKline, Sanofi, Boehringer Ingelheim, Eisai
- Consulting or advisory role: Boehringer Ingelheim, Roche/Genentech, Novartis, GlaxoSmithKline, Eisai, Pfizer, AstraZeneca, Libbs, MSD Oncology, United Medical
- Research funding: Pfizer, Novartis, Amgen, AstraZeneca, Boehringer Ingelheim, GlaxoSmithKline, Roche/Genentech, Lilly, Sanofi, Taiho Pharmaceutical, Mylan, Merrimack, Merck, AbbVie, Astellas Pharma, Biomarin, Bristol-Myers Squibb, Daiichi Sankyo, Abraxis BioScience, AB Science, Asana Biosciences, Medivation, Exelixis, ImClone Systems, LEO Pharma, Millennium, Janssen, Atlantis Clinica, INC Research, Halozyme, Covance, Celgene, inVentiv Health
- Travel, accommodations, expenses: Roche/Genentech, Novartis, Pfizer, BMS Brazil, AstraZeneca, MSD Oncology

## Figures and Tables

**Table 1.** Definitions and applicability of statistic and epidemiologic terms used in this article.

Indicator	Definition	Applicability for this article
MIR	Mortality of a disease divided by its incidence.	It is a proxy for **Lethality**. Example: MIR of 0.2 = Lethality of 20%
GDPpc	Total production of one country divided by its population. It has the same value of income per capita.	It is a proxy for the **Resources Available**.
Residues	Differences between the estimated values by the equations and the real values. In the graphic, it is the measure of the distance of a single point to the regression line.	Measures the **Cost-Effectiveness** of the management of cancer by one country. A positive value means that the Lethality of a cancer site in one country is higher than expected, given its income per capita.
Elasticity	Variable’s sensitivity to the change in another variable. In the graphic, it is the inclination of the regression line.	Measures the decrease in the **Lethality** of each cancer when the **Income** increases 100%.
R-squared	Measure of how the variation of one variable can be explained by another variable’s variation. In the graphic, it is the measure of the distance of all the points from the regression line.	Measures how much of the difference in the **Lethality** of one primary cancer between countries can be explained by the variation of **Income**.

**Table 2.** Results of the regressions for each primary site showing the Brazilian real lethality (calculated from the MIR), the predicted lethality by the model and the differences between these results (which are called residues). Positive residues indicate that the lethality of cancer is higher than it should be, based on its income per capita.

Site	Brazilian real lethality	Brazilian predicted lethality	Difference (residue)
Breast	20%	29%	−9%
Lung	92%	85%	6%
Colon	52%	51%	1%
Prostate	18%	33%	−15%
Cervical	47%	45%	2%

**Table 3.** Results of elasticity and R-squared values for the regressions of each primary site.

Site	Elasticity	R-squared
Breast	5.7%	75%
Lung	4.1%	47%
Colon	6.3%	57%
Prostate	8.5%	71%
Cervix	8.4%	78%

## Appendix

The classification defined by the World Bank for 2018 of Low, Lower-middle, Upper-middle and High-income has the following thresholds [5]:

**Table:**

Threshold	Gross national income/capita (current US$)
Low-income	
Lower-middle income	1,026–3,995
Upper-middle income	3,995–12,375
High-income	>12,375

We present in the Appendix the data used for each primary site (Tables 1a–5a). GDP data was extracted from the World Bank database. Cancer incidence and mortality was extracted from the International Agency for Research on Cancer (an agency of the WHO). Only countries with incidence and mortality with an uncertainty level lower than 15% were considered to give more confidence to the data.

We also present the equations generated by the OLS models for each cancer primary site (Table 6a).

**Table 1a.** Data for breast cancer.

Country name	GDPpc	Breast cancer incidence	Breast cancer mortality
United Arab Emirates	40,698.84934	52.9	16.9
Argentina	14,398.35877	73	18
Australia	53,793.53726	94.5	12.3
Austria	47,380.82964	71.1	14.3
Azerbaijan	4,135.138601	32.7	14.6
Belgium	43,467.4459	113.2	16.3
Bulgaria	8,228.01157	59.1	16.4
Bosnia and Herzegovina	5,148.208517	45.4	14.6
Belarus	5,733.30721	50.4	12.9
Brazil	9,812.278531	62.9	13
Bhutan	3,130.233543	5	2.7
Canada	44,870.77616	83.8	12.1
Switzerland	80,342.84634	88.1	12.3
Chile	15,346.4497	40.9	11.1
China	8,826.994096	36.1	8.8
Colombia	6,408.920012	44.1	11.9
Costa Rica	11,677.26904	46.7	12.2
Cuba	8,433.092699	44.2	14.5
Germany	44,665.50637	85.4	15.7
Denmark	57,218.85196	88.8	14.7
Algeria	4,055.247211	55.6	16.2
Ecuador	6,273.488892	31.8	9.1
Egypt, Arab Rep.	2,412.727082	52.4	21.3
Spain	28,208.30041	75.4	10.6
Ethiopia	767.5634778	41.2	22.9
Finland	45,804.65421	89.5	11.3
France	38,484.18992	99.1	15.4
United Kingdom	39,953.57306	93.6	14.4
Ghana	2,046.109986	43	17.7
Greece	18,885.47598	69.3	13.5
Croatia	13,386.51286	68.7	18.2
Hungary	14,278.8745	85.5	17.9
Indonesia	3,846.415709	42.1	17
India	1,979.364301	24.7	13.4
Ireland	68,885.45038	90.3	17.6
Iran, Islamic Rep.	5,593.853783	31	8.7
Iraq	5,017.968065	38.4	13.6
Italy	32,110.02726	92.8	13.8
Japan	38,430.29124	57.6	9.3
Kazakhstan	9,030.318806	37.2	14.8
Kenya	1,594.834926	40.3	17.8
Lebanon	8,808.589448	97.6	25.3
Sri Lanka	4,073.736519	22.2	8.1
Lithuania	16,809.64826	59.6	16
Latvia	15,684.55852	62.8	17.7
Morocco	3,022.92792	51	17.6
Maldives	11,151.06921	41.2	16.6
Mexico	8,910.333177	39.5	9.9
Malaysia	9,951.544153	47.5	18.4
Nigeria	1,968.425523	41.7	18.8
Netherlands	48,482.76621	105.9	16.5
Norway	75,704.2487	87.5	11
New Zealand	42,583.08473	92.6	14.2
Pakistan	1,547.853414	43.9	23.2
Peru	6,571.928645	40	10.3
Philippines	2,988.952703	52.4	17.5
Poland	13,863.54842	59.1	15.8
Portugal	21,291.43121	70.7	11.3
Qatar	63,249.42243	42.1	13.9
Romania	10,819.24402	51.6	14.6
Russian Federation	10,749.05607	53.6	15.1
Singapore	57,714.29663	64	18.5
Sierra Leone	499.5291033	43.6	25.4
Serbia	5,901.223013	75.3	21.9
Slovenia	23,601.40278	68.5	13.4
Sweden	53,253.47664	89.8	11.4
Togo	610.1517301	29	14.4
Thailand	6,595.004125	35.7	10.9
Tunisia	3,464.41689	32.2	10.3
Ukraine	2,639.824326	44.6	16.7
Uruguay	16,245.59837	65.2	20.7
United States	59,927.93029	84.9	12.7
Uzbekistan	1,533.852038	22.6	11.8
Yemen, Rep.	1,106.803906	24.9	12.7
South Africa	6,151.077955	49	16.3

**Table 2a.** Data for lung cancer.

Country name	GDPpc	Lung cancer incidence	Lung cancer mortality
Albania	4,537.579056	22	19
Argentina	14,398.35877	18.9	17.1
Armenia	3936.79832	29.2	27.2
Australia	53,793.53726	26.2	16.8
Austria	47,380.82964	27.8	21.8
Azerbaijan	4,135.138601	12.8	12
Belgium	43,467.4459	39	27.1
Bulgaria	8,228.01157	28.8	25.7
Bosnia and Herzegovina	5,148.208517	36.1	30.5
Belarus	5,733.30721	25.3	17.2
Bolivia	3,393.955818	7.7	7.2
Brazil	9,812.278531	13	12
Bhutan	3,130.233543	7.9	7.3
Canada	44,870.77616	30	23.5
Switzerland	80,342.84634	22.6	17.2
Chile	15,346.4497	13.4	12.3
China	8,826.994096	35.1	30.9
Colombia	6,408.920012	10.1	9
Costa Rica	11,677.26904	6.6	5.5
Cuba	8,433.092699	31.1	25.8
Germany	44,665.50637	33.7	23.8
Denmark	57,218.85196	36.6	27.6
Dominican Republic	7,052.258839	12.4	11
Algeria	4,055.247211	10.1	10
Ecuador	6,273.488892	6.1	5.7
Egypt, Arab Rep.	2,412.727082	7.6	7.2
Spain	28,208.30041	27	21.2
Estonia	20,200.37559	29.6	23.7
Finland	45,804.65421	19.3	15.6
France	38,484.18992	36.1	26.3
United Kingdom	39,953.57306	32.5	22.2
Georgia	4,045.418967	17.3	16.1
Greece	18,885.47598	40.5	31.8
Guam	35,675.79417	37.9	35.7
Honduras	2,480.125929	5.7	5.2
Croatia	13,386.51286	32.5	30.5
Hungary	14,278.8745	56.7	44.4
Indonesia	3,846.415709	12.4	10.9
India	1,979.364301	5.4	5
Ireland	68,885.45038	33.7	22.1
Iran, Islamic Rep.	5,593.853783	9.1	8.3
Iraq	5,017.968065	10.7	10.4
Israel	40,543.58417	21.1	18.2
Italy	32,110.02726	24.4	19.2
Jordan	4,129.751664	18.4	16.8
Japan	38,430.29124	27.5	16.2
Kazakhstan	9,030.318806	21.6	19.4
Korea, Rep.	29,742.83886	27.8	18.1
Lebanon	8,808.589448	23.2	21
Sri Lanka	4,073.736519	5.1	4.2
Lithuania	16,809.64826	26.6	21.7
Latvia	15,684.55852	25.9	21.2
Morocco	3,022.92792	17.2	17
Maldives	11,151.06921	12.9	11.3
Mexico	8,910.333177	5.8	4.9
Mongolia	3,717.473389	19.6	17.4
Malaysia	9,951.544153	15.3	13.3
Netherlands	48,482.76621	33.3	26.5
Norway	75,704.2487	29.9	20.3
New Zealand	42,583.08473	25.3	18.1
Pakistan	1,547.853414	7.1	6.7
Peru	6,571.928645	9.1	8
Philippines	2,988.952703	21.2	19.2
Poland	13,863.54842	36.5	33.2
Portugal	21,291.43121	22.6	19
Romania	10,819.24402	29.8	26.3
Russian Federation	10,749.05607	24	21
Singapore	57,714.29663	28.6	26.1
El Salvador	3,889.308769	5.5	5.2
Serbia	5,901.223013	49.8	39.9
Slovenia	23,601.40278	32.9	27
Sweden	53,253.47664	17.4	14.9
Thailand	6,595.004125	20.4	18.7
Turkmenistan	6,586.625863	9.2	8.5
Tunisia	3,464.41689	13.9	13.3
Turkey	10,546.15256	36.9	35.9
Ukraine	2,639.824326	20.6	18.5
Uruguay	16,245.59837	27.8	24.8
United States	59,927.93029	35.1	22.1
Uzbekistan	1,533.852038	8.5	7.4
Vietnam	2,342.244003	21.7	19
South Africa	6,151.077955	17.3	16.4

**Table 3a.** Data for colon cancer.

Country name	GDPpc	Colon cancer incidence	Colon cancer mortality
United Arab Emirates	40,698.84934	12.6	6.3
Argentina	14,398.35877	19.6	10.7
Australia	53,793.53726	23.6	3.2
Austria	47,380.82964	12.9	5.8
Azerbaijan	4,135.138601	4.9	3.4
Belgium	43,467.4459	23.1	7
Bulgaria	8,228.01157	16.3	11.1
Bosnia and Herzegovina	5,148.208517	11.8	6.1
Belarus	5,733.30721	19.2	8.8
Brazil	9,812.278531	10.6	5.6
Canada	44,870.77616	18.7	6.8
Switzerland	80,342.84634	14.2	5.3
Chile	15,346.4497	12.7	7.3
China	8,826.994096	12.9	6.3
Colombia	6,408.920012	9.8	5.7
Costa Rica	11,677.26904	10.5	6.3
Cuba	8,433.092699	13	9.1
Germany	44,665.50637	15.7	6.3
Denmark	57,218.85196	25.2	8.9
Algeria	4,055.247211	8.1	4.3
Ecuador	6,273.488892	7.1	4.4
Egypt, Arab Rep.	2,412.727082	4.2	2.5
Spain	28,208.30041	21	8.7
Finland	45,804.65421	15.4	5.4
France	38,484.18992	18.5	7.2
United Kingdom	39,953.57306	20.2	5.9
Greece	18,885.47598	20.8	8.3
Croatia	13,386.51286	22	11.9
Hungary	14,278.8745	32.6	13.6
Indonesia	3,846.415709	6.2	3.9
India	1,979.364301	2.2	1.5
Ireland	68,885.45038	21.4	5.2
Iran, Islamic Rep.	5,593.853783	8.6	4.9
Iraq	5,017.968065	4.2	2.4
Israel	40,543.58417	14	7.4
Italy	32,110.02726	20.6	7.6
Japan	38,430.29124	24.3	7.8
Kazakhstan	9,030.318806	8.1	5
Korea, Rep.	29,742.83886	24.7	5.1
Lebanon	8,808.589448	15	8.2
Sri Lanka	4,073.736519	2	1.1
Lithuania	16,809.64826	15.9	7.1
Latvia	15,684.55852	17.5	6.9
Moldova	2,290.23515	17.6	9.4
Maldives	11,151.06921	6.5	3.7
Mexico	8,910.333177	7.9	4.2
Malaysia	9,951.544153	11.4	6.9
Netherlands	48,482.76621	25.3	9.9
Norway	75,704.2487	27.8	9.4
New Zealand	42,583.08473	22.3	7.6
Peru	6,571.928645	8.8	5.2
Philippines	2,988.952703	12.4	7.6
Poland	13,863.54842	15.9	9.8
Portugal	21,291.43121	21.7	9.6
Romania	10,819.24402	14.5	8.3
Russian Federation	10,749.05607	15.3	8.4
Singapore	57,714.29663	23.6	10.7
Serbia	5,901.223013	17.7	9.2
Slovenia	23,601.40278	26	7.4
Sweden	53,253.47664	17.1	6.8
Thailand	6,595.004125	8	4.5
Tunisia	3,464.41689	6.8	3.8
Turkey	10,546.15256	11.8	8.1
Ukraine	2,639.824326	14	7.9
Uruguay	16,245.59837	24.6	11.8
United States	59,927.93029	15.9	6.3
Vietnam	2,342.244003	5	2.8
South Africa	6,151.077955	9.1	5.3

**Table 4a.** Data for prostate cancer.

Country name	GDPpc	Prostate cancer incidence	Prostate cancer mortality
Argentina	14,398.35877	42.4	12.3
Australia	53,793.53726	85.6	10
Austria	47,380.82964	61.6	9.5
Belgium	43,467.4459	65.5	8.7
Bulgaria	8,228.01157	53.6	12.5
Bosnia and Herzegovina	5,148.208517	26.3	11.5
Belarus	5,733.30721	49.4	13.6
Bolivia	3,393.955818	34.2	9.1
Brazil	9,812.278531	74	13.6
Canada	44,870.77616	58.2	7.8
Switzerland	80,342.84634	77.4	11.1
Chile	15,346.4497	51.2	15.8
China	8,826.994096	9.1	4.7
Colombia	6,408.920012	49.8	12
Cuba	8,433.092699	48.6	22.3
Germany	44,665.50637	63.2	11.3
Denmark	57,218.85196	75.9	15.8
Dominican Republic	7,052.258839	60.1	28
Algeria	4,055.247211	13	4.5
Ecuador	6,273.488892	38.8	13.9
Spain	28,208.30041	73.1	7.4
Finland	45,804.65421	71.6	10.8
France	38,484.18992	99	8.1
United Kingdom	39,953.57306	80.7	12.7
Greece	18,885.47598	50.5	8.8
Guatemala	4,470.989572	39.9	13.2
Croatia	13,386.51286	54.5	15.5
Hungary	14,278.8745	60.2	11.8
India	1,979.364301	4.4	2.9
Ireland	68,885.45038	132.5	11.4
Iran, Islamic Rep.	5,593.853783	16.6	8.3
Iraq	5,017.968065	6.6	2
Italy	32,110.02726	61.3	6
Japan	38,430.29124	35.4	4.4
Kazakhstan	9,030.318806	12.8	7
Korea, Rep.	29,742.83886	36.2	4.7
Lebanon	8,808.589448	39.3	11.9
Sri Lanka	4,073.736519	4	2.2
Lithuania	16,809.64826	70.2	18.1
Latvia	15,684.55852	80.3	21
Morocco	3,022.92792	22.7	10.9
Maldives	11,151.06921	10.4	6.6
Mexico	8,910.333177	41.6	10
Malaysia	9,951.544153	12.4	5.6
Nigeria	1,968.425523	32.8	16.3
Netherlands	48,482.76621	68.9	11.7
Norway	75,704.2487	106.5	16.1
New Zealand	42,583.08473	90.8	11.6
Pakistan	1,547.853414	6.7	5
Panama	15,196.39734	60.7	15.4
Peru	6,571.928645	47.8	15.6
Philippines	2,988.952703	22.9	12.6
Poland	13,863.54842	43.7	14.5
Portugal	21,291.43121	59.5	10.6
Romania	10,819.24402	30.5	10.8
Russian Federation	10,749.05607	39.4	13.6
Singapore	57,714.29663	64.1	8.1
Sierra Leone	499.5291033	29	20
Serbia	5,901.223013	35.4	13.1
Slovenia	23,601.40278	79.3	14.4
Sweden	53,253.47664	103	15
Togo	610.1517301	20.4	13.1
Tunisia	3,464.41689	12.3	5.7
Ukraine	2,639.824326	32	13.8
Uruguay	16,245.59837	59.6	17.1
United States	59,927.93029	75.7	7.7
South Africa	6,151.077955	68	27.9

**Table 5a.** Data for cervical cancer.

Country name	GDPpc	Cervical incidence	Cervical mortality
Argentina	14,398.35877	16.7	7.7
Australia	53,793.53726	6	1.7
Azerbaijan	4,135.138601	6.5	4.6
Bolivia	3,393.955818	38.5	19
Brazil	9,812.278531	12.2	5.8
Bhutan	3,130.233543	14.4	10.2
Canada	44,870.77616	5.7	1.7
Chile	15,346.4497	12.2	5
China	8,826.994096	10.7	4.4
Colombia	6,408.920012	12.7	5.7
Cuba	8,433.092699	14.6	6
Germany	44,665.50637	7.5	2.2
Algeria	4,055.247211	8.1	5.5
Ecuador	6,273.488892	17.8	9
Spain	28,208.30041	5.2	1.7
France	38,484.18992	6.7	2.3
United Kingdom	39,953.57306	8.4	1.7
Ghana	2,046.109986	32.9	23
Guatemala	4,470.989572	21.1	11.7
Honduras	2,480.125929	19.6	12.5
Hungary	14,278.8745	17.2	5.1
Indonesia	3,846.415709	23.4	13.9
India	1,979.364301	14.7	9.2
Iran, Islamic Rep.	5,593.853783	2.2	1.2
Italy	32,110.02726	7.1	1.5
Japan	38,430.29124	14.7	2.7
Kazakhstan	9,030.318806	15.7	7.5
Kenya	1,594.834926	33.8	22.8
Korea, Rep.	29,742.83886	8.4	2
Sri Lanka	4,073.736519	7.8	4.2
Morocco	3,022.92792	17.2	12.6
Maldives	11,151.06921	23.2	13.4
Mexico	8,910.333177	11	5.8
Mali	827.0064008	43.9	36.2
Mozambique	426.2219619	42.8	35.7
Malaysia	9,951.544153	10.5	6
Nigeria	1,968.425523	27.2	20
Peru	6,571.928645	23.2	10.2
Philippines	2,988.952703	14.9	8.8
Poland	13,863.54842	9.4	4.9
Romania	10,819.24402	19.5	8.9
Russian Federation	10,749.05607	17	6.2
Sierra Leone	499.5291033	13.8	12
El Salvador	3,889.308769	18.5	9.4
Serbia	5,901.223013	20.3	7
Togo	610.1517301	23.8	18.7
Thailand	6,595.004125	16.2	9
Turkey	10,546.15256	4.8	2.5
Uganda	606.4684535	54.8	40.5
Ukraine	2,639.824326	17	6.6
United States	59,927.93029	6.5	1.9
Uzbekistan	1,533.852038	9.9	5.4
Vietnam	2,342.244003	7.1	4
South Africa	6,151.077955	43.5	19.2
Zimbabwe	1,333.395663	62.3	46

**Table 6a.** Equations generated by the regression of each primary site. For all of them, the GDPpc coefficients were negative and statistically significant, indicating a negative correlation between income and lethality of cancer.

Breast	MIR = 1.05–0.082.ln (GDPpc)
Lung	MIR = 1.41–0.060.ln (GDPpc)
Colon	MIR = 1.36–0.091.ln (GDPpc)
Prostate	MIR = 1.46–0.123.ln (GDPpc)
Cervix	MIR = 1.58–0.122.ln (GDPpc)
